# Molecular surveillance of drug-resistance associated mutations of *Plasmodium falciparum *in south-west Tanzania

**DOI:** 10.1186/1475-2875-6-2

**Published:** 2007-01-15

**Authors:** Mirjam Schönfeld, Isabel Barreto Miranda, Mirjam Schunk, Ibrahim Maduhu, Leonard Maboko, Michael Hoelscher, Nicole Berens-Riha, Andrew Kitua, Thomas Löscher

**Affiliations:** 1 Department of Infectious Diseases and Tropical Medicine, Ludwig-Maximilians- University (LMU), Munich, Germany; 2 Department of Paediatrics and Child Health, Mbeya Referral Hospital, Tanzania; 3 Mbeya Medical Research Programme (MMRP), Mbeya, Tanzania; 4 National Institute for Medical Research (NIMR), Dar es Salaam, Tanzania

## Abstract

**Background:**

In Tanzania, drug-resistant malaria parasites are an increasing public health concern. Because of widespread chloroquine (CQ) resistance Tanzania changed its first line treatment recommendations for uncomplicated malaria from CQ to sulfadoxine-pyrimethamine (SP) in 2001. Loss of SP sensitivity is progressing rapidly. SP resistance is associated with mutations in the dihydrofolate reductase (*pfdhfr*) and dihydropteroate synthase (*pfdhps*) genes.

**Methods:**

In samples from 86 patients with uncomplicated *Plasmodium falciparum *malaria from Mbeya and Matema, Mbeya region, south-western Tanzania, the occurrence of mutations was investigated in the *pfcrt *and *pfmdr1 *genes which are associated with CQ resistance and in *pfdhfr *and *pfdhps*, conferring SP resistance, as well in *cytb *which is linked to resistance to atovaquone.

**Results:**

*Pfcrt *T76 occurs in 50% and *pfmdr1 *Y86 in 51.7%. *Pfdhfr *triple mutations coexisting with *pfdhps *double mutations were detected in 64.3% of the *P. falciparum *isolates. This quintuple mutation is seen as a possible predictive molecular marker for SP treatment failure. Mutations of the *cytb *gene were not detected.

**Conclusion:**

These findings of a high prevalence of mutations conferring SP resistance correspond to data of *in vivo *SP efficacy studies in other regions of Tanzania and underline the recommendation of changing first-line treatment to artemisinin-based combination therapy.

## Background

In Sub-Saharan Africa malaria is a leading cause of morbidity and mortality, especially in children under five years [1]. Despite intensive campaigns, over 10 Million malaria cases occurred in Tanzania in 2003, about 30% more compared to the previous year [2]. Most of these cases were caused by *Plasmodium falciparum*. This may be partly due to better surveillance systems, but raising drug resistance is the most likely reason for this tremendous increase.

Chloroquine (CQ) was the antimalarial treatment of choice during the second half of the 20^th ^century. But increasing rates of CQ resistance led Tanzania to change its first line treatment of uncomplicated malaria to sulfadoxine-pyrimethamine (SP) in 2001 [3]. This antifolate combination seemed to be an effective and reasonable alternative, but resistance to SP was rapidly gaining ground, facilitated by the slow elimination from the body. New data show a high level (45%) of SP treatment failures in Muheza, northeast Tanzania [4]. Other effective drugs, where resistance is as yet not a frequent problem, such as atovaquone-proguanil or mefloquine, are of limited value due to their high current costs [5]. A useful alternative is artemisinin-based combination therapy (ACT), e.g., artemether-lumefantrine which is introduced as the new first line drug in Tanzania in mid 2006. At the moment the availability of ACT is limited; while artemisinin monotherapy formulations and SP are commonly used, especially in the private sector. Some drug shops still sell the relegated CQ.

CQ resistance is associated with an amino acid change from lysine to threonine in codon 76 of the *P. falciparum *chloroquine resistance transporter gene (*pfcrt*) [6], and a mutation from asparagine to tyrosine in codon 86 of the multidrug resistant gene (*pfmdr*) [7-10]. Besides there are indices that *pfmdr1 *N86 is associated with resistance to lumefantrine that is widely used in combination with artemether [11], and also with decreased sensitivity to artemisinins [12].

SP resistance is associated with mutations in the dihydrofolate reductase (*pfdhfr*) and dihydropteroate synthase (*pfdhps*) genes. Pyrimethamine is a selective, competitive inhibitor of dihydrofolate reductase and earlier in the folate pathway sulfa drugs inhibit dihydropteroate synthase [13,14]. Several point mutations are connected to antifolate drug resistance. The quintuple mutation (triple *pfdhfr*: I51, R59, N108 and double *pfdhps*: G437, E540) is discussed as a relevant molecular marker of SP treatment failure [15,16].

Atovaquone-proguanil is a relatively new antimalarial drug that inhibits mitochondrial electron transport. Point mutations in the *cytb *codon 268 are associated with resistance to this combination [17-19].

## Methods

Overall 86 blood samples were collected from patients with clinically diagnosed uncomplicated *P. falciparum *malaria (fever ≥ 38.0°C, parasitaemia ≥ 2 000/μl) from August to October in 2004 and from June to July in 2005 in Mbeya region of South-western Tanzania. Patients were enrolled at two locations: Matema Health Care Center located in a poor rural area at the shores of Lake Malawi with holoendemic malaria transmission and the Mbeya Referral Hospital, a tertiary hospital at an altitude of 1 700 meters that admits complicated cases from surrounding mesoendemic areas or people that have travelled within Tanzania. Written informed consent was obtained from each patient or the parental guide. The study was reviewed and approved by the local IRB at the Mbeya Referral Hospital and the Tanzanian National Ethics Board of the National Institute for Medical Research, Dar es Salaam.

From each patient a finger prick blood sample was taken for thick and thin blood film and another for filter paper blood sample. Giemsa-stained blood films were examined for malaria parasites (per 200 white blood cells) and densities were assessed based on a assumed mean WBC count of 8 000/μl. DNA extraction from filter paper bloodspots was done using Chelex^® ^(Bio-Rad, Germany) as described elsewhere [20] Nested PCR assays were used to verify the parasite species [21]. The DNA was amplified by nested PCR and digested by the RFLP-method to detect the mutations of *P. falciparum pfcrt *76, *pfmdr1 *86, *dhfr *16, 51, 59, 108, 164, *dhps *436, 437, 540, 581, 613 and *cytb *268 [17,22,23]. Mixed alleles (wild type and mutant) were assessed as mutant. The median age of the 86 patients with *P. falciparum *infection (45 female, 41 male) was 21 years (range 8 month to 55 years). All 86 isolates showed *P. falciparum *mono-infection, there was no *P. vivax*, *P. ovale*, or *P. malariae *infection. The geometric mean parasite density was 29 992/μl (range 3 320/μl to 127 440/μl).

## Results

Table 1 displays the prevalence of *pfcrt*, *pfmdr1*, *pfdhfr*, *pfdhps *and *cytb *alleles in Mbeya & Matema. *Pfcrt *T76 mutation is expressed by 58.8% in Mbeya and 47.8% in Matema. 70.6% of the isolates from Mbeya and 47.1% from Matema showed the *pfmdr1 *Y86 mutation. Almost 100% of both settings exhibited the *pfdhfr *N108 mutation. Likewise, nearly all samples showed the I51 mutation. All but one displayed the R59 mutation in Mbeya. Amino acid changes from alanine to glycine at codon 437 and from lysine to glutamine at codon 540 of the *pfdhps *gene were detected in 81% and 86.9%, respectively. Only one specimen in Matema displayed the G581 variant. No *pfdhfr *L164 mutation was seen. T108 and V16 variants which are linked with cycloguanil resistance were not present as well. In line with previous reports no evidence for *cytb *codon 268 mutations were found in south-western Tanzania [24].

**Table 1:** Prevalence of mutations conferring resistance to chloroquine, sulfadoxine-pyrimethamine and atovaquone-proguanil in *Plasmodium falciparum *isolates from Mbeya & Matema, southern Tanzania

**Gene**	**Mutation**	**N**	**Mutation (%)**	**Mixed type (%)**
*Pfcrt*	T76	86	37 (43)	6 (7)
*Pfmdr1*	Y86	85	38 (44.7)	6 (7.1)
				
*Dhfr*	I51	86	80 (93)	0 (0)
	N108	86	84 (97.7)	0 (0)
	T108	86	0 (0)	0 (0)
	R59	86	69 (80.2)	6 (7)
	V16	86	0 (0)	0 (0)
	L164	86	0 (0)	0 (0)
				
*Dhps*	A436	84	4 (4.8)	3 (3.6)
	G437	84	66 (78.6)	2 (2.4)
	E540	84	65 (77.4)	8 (9.5)
	G581	81	1 (1.2)	0 (0)
	N613	81	0 (0)	0 (0)
				
*Cytb*	N268	85	0 (0)	0 (0)

## Discussion

More relevant for predicting treatment failure or emerging resistance are combinations of the point mutations described above (Table 2). In Matema, the rural setting, the *Pfdhfr *quintuple mutation was more common than in the Mbeya Referral Hospital (67.2% vs 52.9%), while the *Pfdhfr *triple mutation, suggested to be an early molecular marker for SP resistance in Tanzania [25], was more frequent in Mbeya (79.7% vs 94.1%). Although the predictive value of these markers for SP treatment failure has not been established in the study regions, these results are in line with the high level of treatment failure (42.3%) in a multi-site survey in Tanzania [26].

**Table 2:** Prevalence of *pfcrt*, *pfmdr*1, *dhfr *and *dhps *genotype combinations conferring chloroquine and sulfadoxine-pyrimethamine resistance in Mbeya & Matema, together and each seperated. The risk ratio is the prevalence ratio of the combinations between Mbeya and Matema.

**Grouped alleles**					
		**Mbeya & Matema (%)**	**Mbeya (%)**	**Matema (%)**	**Risk ratio**
*Pfcrt*+*pfmdr1*	T76+Y86**	29 (34.1)	9 (52.9)	20 (29.4)	0.5556
*Dhfr*	I51+N108*	80 (93)	17 (100)	63 (91.3)	0.9130
	I51+R59*	71 (82.6)	16 (94.1)	55 (79.9)	0.8469
*Dhfr *triple	I51+R59+ N108*	71 (82.6)	16 (94.1)	55 (79.7)	0.8469
*Dhps *double	G437+E540***	68 (81)	10 (58.8)	58 (86.6)	1.4716
*Dhfr/dhps *quintuple (triple *dhfr *+ double *dhps*) ***		54 (64.3)	9 (52.9)	45 (67.2)	1.2687

* N = 86 ** N = 85 *** N = 84

Even so this study investigated a limited number of patients and therefore differences may not reach significance, differences in mutation rates might reflect differences of access to health care between the two locations. While individuals in the rural area receive their malaria treatment, currently SP, almost exclusively through the Matema Health Care Center, patients from Mbeya can choose upon a wide variety of health care facilities and antimalarial drugs. Ongoing usage of CQ may be assumed there.

Although considerably higher resistance rates before 2001 are probable, the still relatively high rate of *pfcrt *and *pfmdr1 *mutations is contrary to other reports that have demonstrated a complete regression of *pfcrt *mutations several years after leaving CQ as first – line antimalarial drug [27,28].

In vivo selection of *pfmdr1 *86N allele by artemether-lumefantrine has been found in Tanzania [11] and *pfmdr *copy numbers seem to influence susceptibility to lumefantrine and artemisinin [29]. There is no prediction possible due to our results but continuing surveillance would be interesting concerning *pfmdr1 *polymorphisms.

## Conclusion

This study confirms the high prevalence of point mutations in the *pfdhfr *and *pfdhps *genes in Tanzania which are associated with SP treatment failure [25,26]. The rate of quintuple *pfdhfr/pfdhps *mutations in the Mbeya region, south-western Tanzania, is in the upper range of frequencies reported in East Africa. Data from Malawi, Kenya, Tanzania and Ethiopia range from 10 to 78% [25,30-32]. The absence of *cytb *codon 268 supports atovaquone-proguanil as a possible second- or third-line drug for treatment of uncomplicated malaria.

The study data might be used as a basis for surveillance of resistance markers after introduction of ACT and might later indicate the possibility for reintroduction of one of the other drugs.

## Authors' contributions

LM, AK, MH and TL designed the study. MS, IM and LM were responsible for patient recruitment and parasitological examinations. MS, IBM and MS did the PCR assays. MH did the data processing. MS, AK, IBM, MS, NBR and TL wrote the paper with major contributions of the other authors.

## Conflict of interest

The authors do not have a commercial or other association that might pose a conflict of interest.
